# Evaluation of effectiveness of bacteriophage purification methods

**DOI:** 10.1186/s12985-024-02580-y

**Published:** 2024-12-19

**Authors:** Siti Saleha Binte Mohamed Yakob Adil, Joseph Tucci, Helen Irving, Cassandra Cianciarulo, Mwila Kabwe

**Affiliations:** 1 Department of Rural Clinical Sciences, La Trobe Rural Health School, La Trobe University, Bendigo, VIC 3550 Australia; 2 La Trobe Institute of Molecular Science, La Trobe University, P.O Box 199, Bendigo, VIC 3550 Australia; 3 Holsworth Initiative for Medical Research, Rural People, La Trobe Rural Health School, La Trobe University, Bendigo, VIC 3550 Australia

**Keywords:** Bacteriophage, Endotoxin purification, Triton X-100, Caesium chloride, Endotoxin removal resin

## Abstract

The use of bacteriophages for therapy has increased over the last decade. While there is need for clear regulatory pathways for bacteriophage approval for mainstream use in clinical practice, practitioners and patients have been able to access bacteriophage therapy under compassionate grounds and through magistral preparations. However, there is currently no standard for purifying these bacteriophages to ensure safety, and good manufacturing practice certification may not be achieved in these emergency uses. In this study, we employed an Interleukin Receptor Associated Kinase (IRAK) 3 knockout monocyte-based assay to evaluate the endotoxin removal efficacy of three common bacteriophage purification methods: Triton X-100 exposure, CsCl density gradient ultracentrifugation, and Pierce™ High-Capacity Endotoxin Removal Resin spin columns. In our experiments we tested these purification methods on three different bacteriophage morphotypes: siphovirus, podovirus and myovirus. We showed that the lowest endotoxin levels and immune responses were achieved when purifying bacteriophages with Triton-X treatment. The results from purifying with CsCl density gradient ultracentrifugation were comparable, and these were both significantly better than purification with Pierce™ High-Capacity Endotoxin Removal Resin spin columns. We also showed that Triton X-100 purification resulted in the lowest loss of bacteriophage titres. Finally, of the bacteriophages tested here, it did not appear that virus morphology affected efficacy of endotoxin removal.

**Supplementary Information:**

The online version contains supplementary material available at 10.1186/s12985-024-02580-y.

## Introduction

Bacteriophages, independently discovered in 1915 and 1917 by Frederick Twort and Felix d’Herelle, respectively, are viruses that specifically infect bacteria [1]. Their use in modern medicine has been propelled by increased antibiotic resistance and they are considered frontrunner alternatives to antibiotics [2], especially after the declaration of the antibiotic resistance pandemic [3]. Developing bacteriophage therapeutics is considered more affordable than designing new chemical antimicrobial compounds [4]. Further, the capacity for bacteriophages to overcome bacterial resistance through their co-evolutionary capacity is a useful tool for evolving therapeutics [5]. Accordingly, western medicine has seen a significant increase in clinical trials evaluating the safety and efficacy of these viruses [6].

Because bacteriophages are diverse biological entities, procedures to test the safety of each virus need to be employed prior to clinical use. Despite reports of bacteriophage safety in therapy [7], retrospective observational studies have suggested that bacteriophage therapy may have resulted in non-serious adverse events in up to 8% of patients, life-threatening reactions in 1% of patients, and contributed to mortality in 6% of patients [8]. Although bacteriophages and conventional antibiotics are mechanistically different, many experts agree that bacteriophage therapy needs to be proven in the context of randomised controlled trials (RCTs), similar to antibiotics. However, unlike antibiotics, bacteriophages are dynamic and evolve quickly, and may require an outside-the-box regulatory framework for their application [9]. Underestimating RCT and regulation framework requirements for bacteriophages can be costly, as evidenced by the failed ‘Phagoburn’ RCT where inadequate care in preparation of bacteriophages resulted in inactive viruses and ineffective therapy [10]. Presently, it is unclear how bacteriophages may be adapted for use, but examples may be taken from other biologics such as faecal microbiota transplant and how they are regulated [11].

As regulatory discussions progress, bacteriophage therapy is finding its way to patients through compassionate use in many Western hospitals and as magistral preparations in Belgium [9]. In both circumstances, specific bacteria (usually multi-resistant to antibiotics) are isolated from the patient and several bacteriophage biobanks are used to screen for active bacteriophages. If no active bacteriophages are found, bacteriophage hunting is commenced in clinical and research laboratories. The host bacteria strain is cultured for this hunting process and for the production of more bacteriophage particles. Once an active bacteriophage is found, several considerations are taken into account before administration to the patient. Firstly, their genomes are characterised for presence of toxins and antibiotic resistance genes, and then assessed for obligatory lytic capacity. To ensure safety, bacteriophages are purified to remove endotoxins using purification methods in clinical and research laboratories that are often without good manufacturing practice certification [9, 12]. It is therefore reasonable to suggest that safety assurance protocols are required before compassionate and/or magistral applications of bacteriophages in therapy.

The United States Food and Drug Administration (FDA), along with other major regulatory bodies including the Australian Therapeutic Goods Administration and the European Medicines Agency, provide guidelines for maximal endotoxin concentration of parenteral medicines. They place the upper limit at 5 Endotoxin Units (EU) per kg of body weight per hour for non-intrathecal administration, and 2 EU per kg of body weight per hour for intrathecal administration [13]. This is measured by a Limulus Amebocyte Lysate (LAL) test [14] as the gold standard assay. However, as this assay is based on horseshoe crab plasma response [15], it may not reflect or give an indication of expected safety when administered to a patient. Phase I safety trials and case studies have shown variable outcomes in some patients. The adverse events observed can be non-specific and range from local responses such as redness and pain at the application site; mild events such as abdominal discomfort, coughing; life-threatening events such as heart failure; fatal events including septic shock, and bacteriophage therapy associated tumour progression [8]. Further, some methods for removing endotoxins may require researchers to dilute their bacteriophage preparations in order not to exceed recommended FDA limits [16], which may limit bioavailability and efficacy.

There is a paucity of data on the evaluation of endotoxin concentration in bacteriophage preparations. Two studies have compared different bacteriophage purification methods, and used the amoebocyte lysate-based assay to determine endotoxin concentration. One of these compared several sequential bacteriophage purifications by using EndoTrap^®^ HD affinity column followed by CsCl ultracentrifugation or Triton X-100, 1-octanol extraction, enzymatic inactivation of endotoxins and anion-exchange chromatography. Sequential steps of EndoTrap^®^ HD affinity column followed by CsCl ultracentrifugation was found to be the most effective in reducing endotoxin concentration [17]. However, a lack of experimental replicates made it difficult to validate the findings. The other research compared several methods including PEG precipitation/Triton X-100, octanol extraction, anion exchange, and two endotoxin removal columns (EndoTrap^®^ HD affinity column and Pierce™ High-Capacity Endotoxin Removal Resin spin columns). This study observed that using EndoTrap^®^ affinity column in combination with Vivaspin ultrafiltration columns with 100,000 MWCO polyethersulfone membrane was the most effective in removing endotoxin [18]. While the study included data from experimental replicates, diverse bacteriophage morphologies were not directly compared.

In this study, we employed a novel human immune cell-based system for determining concentration of endotoxins in bacteriophage preparations [19] and assessed three common purification techniques: Triton X-100 exposure, Pierce™ High-Capacity Endotoxin Removal Resin spin columns and CsCl density gradient ultracentrifugation [20–22]. We also tested bacteriophages of three different morphotypes to understand whether the viral morphology may affect endotoxin purification.

## Materials and methods

### Bacteriophage preparations and titre assessment

Three bacteriophages were used in this study to represent the three most common morphotypes of the Caudoviricetes. These included Latrobevirus FNU1 (morphotype: siphovirus, genome size 130 kb, GenBank accession number: MK554696), unclassified Ahphunavirus LAh5 (morphotype: podovirus, genome size 42 kb, GenBank accession number: MK838111), and Ludhianavirus LAh10 (morphotype: myovirus genome size 260 kb, GenBank accession number: MK838116). The capsid diameters of these bacteriophages were 82 nm (LAh5), 116 nm (LAh10), and 88 nm (FNU1). The bacteriophages were propagated on their host strains, *Fusobacterium nucleatum* ATCC 10953 for FNU1 [23], and *Aeromonas hydrophila* strains AHB0147 and AHB0116 for LAh5 and LAh10, respectively [24].

*F. nucleatum* was cultured anaerobically at 37 ^o^C using anaerobic generating packs (AnaeroGen™, Oxoid, Australia) in Heart Infusion broth or agar (0.8% w/v) media supplemented with 0.5% cysteine (Sigma, Australia) and 0.5% haemin (Sigma, Australia) while *A. hydrophila* was cultured aerobically at 37 ^o^C in nutrient broth or agar (1% w/v) as previously described [23, 24].

A bacteriophage stock was prepared using the spread technique to increase the volume and concentration. Briefly, 200 µL of bacteriophage stock was spread on a fresh lawn of host bacteria and incubated over 24–48 h, for *A. hydrophila* and *F. nucleatum*, respectively. Bacteriophages were harvested by washing and filtering using 0.2 μm filters (Microanalytix, Australia) and titres calculated using an established method [25]. Filtered bacteriophage stock obtained at this point were considered a crude preparation for this study.

### Bacteriophage purification

Three independently prepared 0.2 μm filtered bacteriophage stocks (normalised to 1 × 10^9^ PFU mL^− 1^) of each bacteriophage type were used in three purification methods. These included the (i) Polyethylene Glycol 8000 (Sigma-Aldrich, Australia) precipitation and Triton X-100 (Sigma-Aldrich, Australia) purification [19], (ii) Caesium chloride (CsCl; Sigma-Aldrich, Australia) density gradient ultracentrifugation [26], and (iii) Pierce™ High-Capacity Endotoxin Removal Resin spin columns (ThermoFisher, Australia) [18]. Bacteriophage titres were assayed before and after purification and stored at -80 ^o^C before endotoxin assessment.

#### PEG precipitation and Triton X-100 purification

Five mL of 0.2 μm filtered bacteriophages were purified as previously described [20, 27]. Briefly, 5 mmol L^− 1^ of MgCl_2_ and 1.0 µL each of RNase A (Promega, Australia) and DNase I (Promega, Australia) to a final concentration of 10 µg mL^− 1^ were added before incubation at room temperature for 30 min to digest extraneous DNA and RNA. Polyethylene glycol 8000 (PEG) at 10% [w/v] and NaCl at 1 g L^− 1^ were added and dissolved by gentle shaking on an orbital mixer (Ratek, Australia) for 5 min. To this mixture, 2% v/v Triton X-100 was added at room temperature, and the material shaken gently for 5 min before a 15 min incubation at 4 ^o^C. After centrifugation (4000 ×*g*; 15 min), the supernatant was discarded and the pellet of precipitated bacteriophages resuspended in 5 mL sterile PBS (phosphate buffered saline, pH 7.4). The PEG/NaCl/Triton X-100 treatment was repeated two more times, following which the bacteriophages were centrifuged (4000 ×*g;* 15 min) and resuspended with fresh sterile 5 mL PBS three times then filtered with a 0.2 μm filter, a precautionary step to ensure that no precipitates are carried forward in the purified bacteriophage solution.

#### Caesium chloride gradient ultracentrifugation

Purification using CsCl was adapted from the method described by Luong and colleagues [26]. Protocol transfer was achieved using the Beckman protocol transfer assistant ‘Intellifuge’ [28]. Briefly, CsCl densities of 1.30 g mL^− 1^, 1.50 g mL^− 1^ and 1.60 g mL^− 1^ were carefully layered in 13.2 mL open-top thin wall ultra-clear centrifuge tubes (Beckman, Australia) in 0.2 μm sterile filtered Tris-sodium chloride buffer, pH 7.0 (10 mM Tris (pH 7.0) and 150 mM NaCl). Adjusting the weight of the centrifuge holders, approximately 4 mL of crude bacteriophage preparation was added. The gradients were centrifuged in a Beckman SW40 Ti rotor at 28,000 ×*g*, 4 °C, for 258 min (Beckman Coulter, Optima L-100 XP Ultracentrifuge). Purified bacteriophages were extracted using a 26-gauge needle and 3 mL syringe from the base of a visible whitish/grey band.

#### Pierce™ high-capacity endotoxin removal resin spin columns

One mL spin columns and endotoxin removal resins were purchased from ThermoFisher, Australia. Before use, resins were equilibrated overnight at room temperature with 8 mL of 0.2 N NaOH to remove storage solution as per manufacturer’s instructions. All solutions are removed from resins by 500 ×*g* centrifugation for 1 min. The NaOH was then replaced with 8 mL of 2 M NaCl, which was replaced with 8 mL of endotoxin-free water and then endotoxin-free buffer. The resins were further washed twice in 8 mL endotoxin-free buffer. After removing endotoxin-free buffer, 5 mL of crude bacteriophage preparations were added to the resin and incubated while mixing at room temperature for 2 h. Purified bacteriophages were eluted by 500 ×*g* centrifugation for 1 min. Resins were then treated with 8 mL of 0.2 N NaOH overnight and stored in 20% ethanol at 4^o^C for subsequent re-use.

### Cytokine detection assay

Interleukin Receptor Activated Kinase (IRAK) 3 knockout THP-1 monocytes were obtained from the La Trobe University collection [29]. The IRAK3 knockout monocytes were cultured in 10% Foetal Bovine Serum (Sigma-Aldrich, Australia) supplemented Roswell Park Memorial Institute (RPMI) 1640 media (Sigma-Aldrich, Australia) at 5% CO_2_ and 37 ^o^C. Approximately 2 × 10^5^ cells per well were seeded in 12-well plates. The cells were exposed to either 300 µL of crude or purified bacteriophage adjusted to 3.0 × 10^8^ PFU mL^− 1^, or PBS, or lipopolysaccharide (LPS) (*Escherichia coli* O55:B5, Sigma; at a concentration of 1 µg/mL) before incubating for 24 h. The supernatant was then collected after centrifugation (300 ×*g*, 5 min, 37 °C) and analysed using BD OptEIA™ Set Human IL-6 (Interleukin 6) and TNF-α (Tumour necrosis factor – α) commercial sandwich Enzyme-Linked Immunosorbent Assays (ELISA; BD Biosciences), according to manufacturer’s instructions.

### Endotoxin correlation

For correlation of cytokine production to endotoxin levels, IRAK3 knockout monocytes were exposed to the 10,000 United States Pharmacopeia Endotoxin Unit Reference Standard diluted from 50 EU mL^− 1^. Endotoxin free water was used as a 0 EU mL^− 1^ (Blank) standard. Cytokine production was analysed as above and standard curves generated by plotting cytokine levels against endotoxin concentrations. Estimated concentration of endotoxin present in the crude and purified bacteriophage samples were calculated by linear regression with cytokine production as the independent variable.

### Statistical analysis

All data were collected into Microsoft Excel spreadsheets before importing to *R/R* studio (R version 4.3.1 (2024.04.2 + 764)). The Shapiro–Wilk test was used to determine whether the data were normally distributed. A paired *t*-test was used for quantitative data that were normally distributed while Wilcoxon signed-rank test was used for non-parametric statistical analysis. A *p *value less than 0.05 was considered statistically significant. Standard curves of endotoxin assay were plotted using the ggscatter function. The correlation factor (*R*) and equation of the linear regression line were calculated using the stat_cor and stat_regline_equation functions.

## Results

### Pro-inflammatory cytokine production in IRAK3 knockout THP-1 cells

**Fig. 1:**
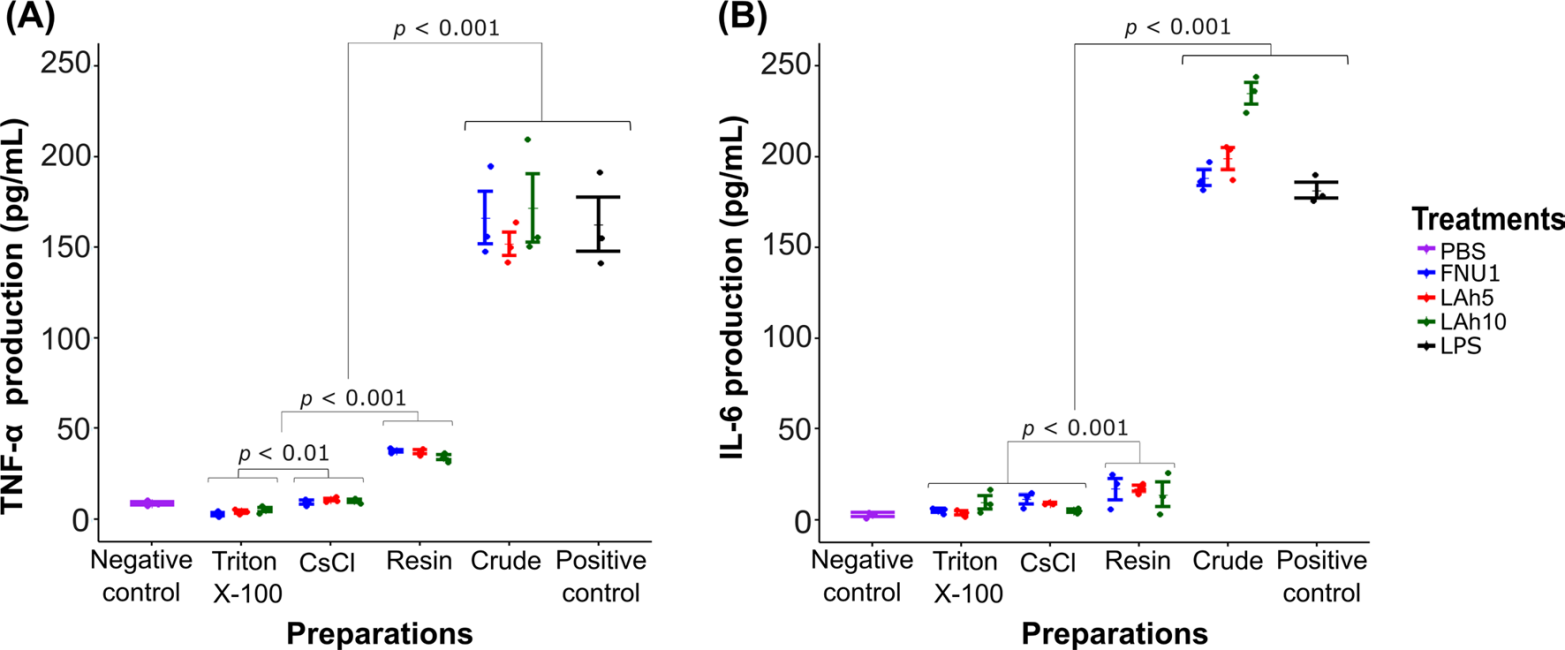
Effects of crude and purified bacteriophages on pro-inflammatory cytokine production in IRAK3 knockout THP-1 cells; **(A)** TNF-α, **(B)** IL-6. Bacteriophages were purified using Triton X-100, CsCl ultracentrifugation and Pierce™ High-Capacity Endotoxin Removal Resin spin columns. IRAK3 knockout cells were stimulated with PBS (negative control) or 1 µg mL^− 1^ LPS (positive control) or crude or purified bacteriophages (3.0 × 10^8^ PFU mL^− 1^). Wilcoxon signed-rank test was used to analyse the data (*n* = 3; error bars indicate standard error of the mean; *p* < 0.05)

Bacteriophages purified via CsCl ultracentrifugation or Pierce™ High-Capacity Endotoxin Removal Resin spin columns produced significantly higher levels of TNF-α and IL-6 (*p* < 0.001) in IRAK3 knockout monocytes (Table 1), compared to those purified using Triton X-100. The production of these pro-inflammatory cytokines by IRAK3 knockout monocytes treated with crude bacteriophage preparations was significantly higher (*p* < 0.001) than those when treated with any of the purified preparations (Fig. 1).

**Table 1:** Pro-inflammatory cytokine production in bacteriophage phage purifications

	Bacteriophage	Cytokine production; Mean pg mL^− 1^ (standard error)	
		TNF-α	IL-6
Crude preparation	FNU1	166.27 (14.4)	188.44 (4.48)
	LAh5	151.89 (6.5)	198.83 (5.82)
	LAh10	171.89 (18.9)	234.74 (5.81)
	Group mean*	163.35 (7.71)	207.33 (7.52)
**Purification method**			
Triton X-100	FNU1	2.70 (1.01)	5.27 (1.00)
	LAh5	4.01 (0.45)	3.79 (1.02)
	LAh10	5.30 (1.17)	9.70 (3.57)
	Group mean	4.01 (0.62)	6.25 (1.42)
CsCl	FNU1	9.41 (1.08)	11.35 (2.60)
	LAh5	10.74 (0.87)	9.05 (0.35)
	LAh10	10.16 (0.81)	4.95 (0.82)
	Group mean*	10.10 (0.50)	8.45 (1.23)
Pierce™ High-Capacity Endotoxin Removal Resin spin columns	FNU1	37.50 (0.87)	16.77 (5.64)
	LAh5	36.93 (1.07)	9.05 (0.35)
	LAh10	34.02 (1.50)	4.95 (0.82)
	Group mean*	36.15 (0.80)	15.90 (2.58)
PBS		8.65 (0.94)	2.98 (1.10)

The means are from 3 independent replicates

*p < 0.001 compared to Triton X-100 purification

### Assay standardisation

**Fig. 2:**
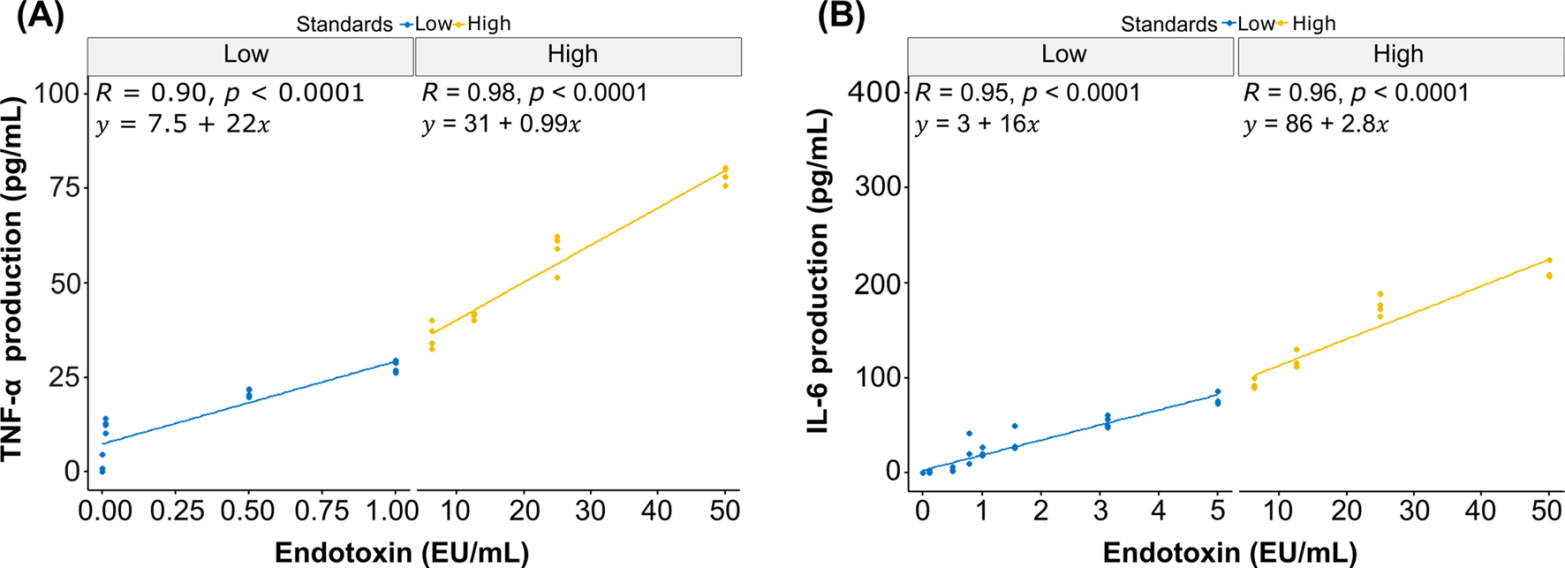
Biphasic standard curves of cytokine production by IRAK3 knockout THP-1 cells, categorised by low and high endotoxin concentrations; **(A)** TNF-α and **(B)** IL-6. Wilcoxon signed-rank test was used to compare the rank of the data medians (*n* = 3; *p-*value < 0.05)

Standard curves were derived from TNF-α and IL-6 production by the IRAK3 knockout THP-1 cells when exposed to known endotoxin standards. Both the standard curves of TNF-α and IL-6 showed a bi-phasic linear correlation between cytokine production and endotoxin standards (Fig. 2). For the TNF-α production, the lower and higher endotoxin standards showed a correlation of *R* = 0.9, *p* < 0.0001 and *R* = 0.98, *p* < 0.0001 respectively. The correlation for the lower and higher endotoxin standards with production of IL-6 was *R* = 0.95, *p* < 0.0001, and *R* = 0.96, *p* < 0.0001, respectively.

### Comparison of the bacteriophage purification methods

**Fig. 3:**
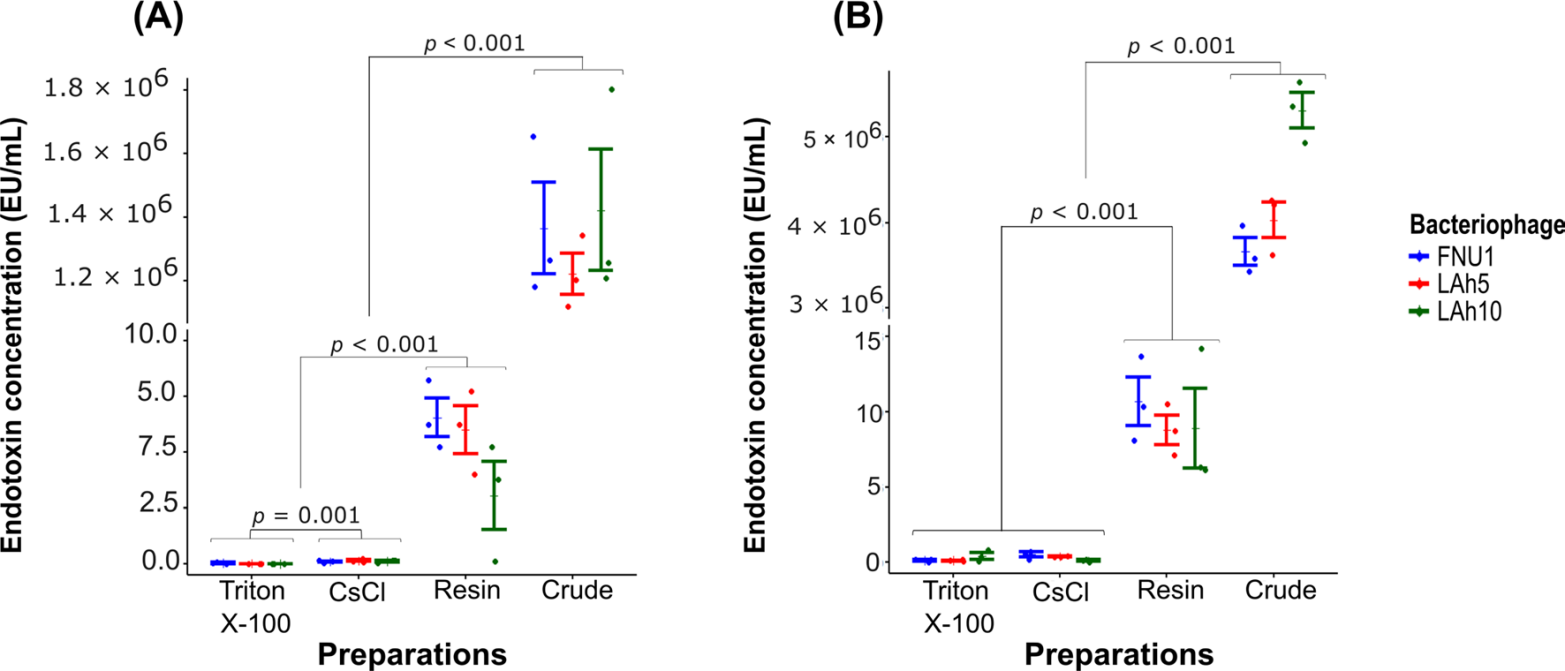
Estimated endotoxin concentrations in bacteriophage preparations were calculated from standard curves: **(A)** TNF-α and **(B)** IL-6. Concentrations of endotoxin in crude and purified bacteriophage preparations were calculated and expressed as EU/mL. Wilcoxon signed-rank test was used to analyse the data (*n* = 3; error bars indicate standard error of the mean; *p-*value < 0.05 is significant)

We found significantly reduced endotoxin concentrations in purified bacteriophages using all three methods, compared to crude preparations (Table 2). The estimated endotoxin concentrations of purified bacteriophage preparations using CsCl ultracentrifugation and Pierce™ High-Capacity Endotoxin Removal Resin spin columns were significantly higher (*p* = 0.001, *p <* 0.001, respectively) than the Triton X-100 bacteriophage preparations, when assessed by measuring TNF-α production in the IRAK3 knockout monocytes (Fig. 3). When assessed by measuring IL-6 cytokine production, the CsCl ultracentrifugation and Triton-X methods of purification were not significantly different, while the Pierce™ High-Capacity Endotoxin Removal Resin spin columns yielded significantly higher endotoxin concentrations (*p <* 0.001) (Fig. 3).

**Table 2:** Estimated levels of endotoxin present in bacteriophage preparations

Purification	Bacteriophage	Endotoxin concentration estimated by cytokine production; Mean EU/mL (Standard Error)	
		TNF-α	IL-6
Crude preparation	FNU1	1,366,322 (145,801.60)	3,658,483 (159,961.2)
	LAh5	1,221,067 (65,256.79)	4,029,466 (207,684)
	LAh10	1,423,178 (190,692.90)	5,312,156 (207,475.90)
	Group mean	1,336,856 (77858)**	4,333,369 (268,435)**
Triton X-100	FNU1	0.04 (0.03)	0.14 (0.06)
	LAh5	0.01 (< 0.01)	0.13 (0.03)
	LAh10	< 0.01 (< 0.01)	0.42 (0.22)
	Group mean	0.02 (0.01)	0.23 (0.08)
CsCl	FNU1	0.10 (0.03)	0.52 (0.16)
	LAh5	0.15 (0.04)	0.38 (0.02)
	LAh10	0.12 (0.04)	0.12 (0.05)
	Group mean	0.12 (0.02)*	0.34 (0.08)
Pierce™ High-Capacity Endotoxin Removal Resin spin columns	FNU1	6.57 (0.88)	10.69 (1.62)
	LAh5	5.99 (1.08)	8.77 (0.99)
	LAh10	3.05 (1.52)	8.89 (2.66)
	Group mean	5.20 (0.80)**	9.45 (0.99)**

The mean values shown are averages are from 3 independent replicates

*p = 0.001 compared to purified Triton X-100

**p < 0.001 compared to purified Triton X-100

### Effect of bacteriophage characteristics on efficacy of purification methods

To assess whether there was a difference in purification efficacy between the bacteriophages, levels of endotoxins measured from standard curves derived from responses of both of the pro-inflammatory cytokines were employed. There was no significant difference between the different bacteriophage types, *p* > 0.05 (Table 3).

**Table 3:** Endotoxin concentrations stratified by morphology

Purification method	Bacteriophage morphology (EU mL^− 1^); Mean (Standard Error)		*p*-value
	**Siphovirus (*n*** **= 6)**	**Podovirus (*n*** **= 6)**	
Triton X-100	0.09 (0.04)	0.07 (0.03)	0.69
CsCl	0.31 (0.12)	0.26 (0.06)	0.43
Endotoxin Removal Resin	8.63 (1.24)	7.38 (0.90)	0.36
	**Siphovirus (*n*** **= 6)**	**Myovirus (*n*** **= 6)**	
Triton X-100	0.09 (0.04)	0.21 (0.14)	0.72
CsCl	0.31 (0.12)	0.12 (0.03)	0.18
Endotoxin Removal Resin	8.63 (1.24)	5.97 (1.89)	0.06
	**Myovirus (*n*** **= 6)**	**Podovirus (*n*** **= 6)**	
Triton X-100	0.21 (0.14)	0.07 (0.03)	0.44
CsCl	0.12 (0.03)	0.26 (0.06)	0.27
Endotoxin Removal Resin	5.97 (1.89)	7.38 (0.90)	0.52

Compared using student’s *t*-test

**Table 4:** Estimated levels of endotoxin present in bacteriophage samples using the Pierce™ high-capacity endotoxin removal Resin spin columns

Purification	Bacteriophage	Endotoxin concentration via cytokine production; Mean EU mL^− 1^ (standard error)	
		TNF-α	IL-6
First passage through resin	FNU1	6.57 (0.88)	10.69 (1.62)
	LAh5	5.99 (1.08)	8.77 (0.99)
	LAh10	4.38 (0.44)	8.89 (2.66)
	Group mean	5.65 (0.53)	9.45 (0.99)
Second passage through resin	FNU1	322.07 (35.99)	49.40 (6.02)
	LAh5	290.71 (34.60)	53.70 (2.60)
	LAh10	273.90 (8.50)	40.07 (4.86)
	Group mean*	295.56 (16.23)	47.72 (3.10)
Third passage through resin	FNU1	444.76 (27.90)	93.02 (4.40)
	LAh5	453.74 (34.60)	101.81 (1.56)
	LAh10	529.49 (10.41)	96.47 (4.86)
	Group mean*	476.00 (16.27)	97.10 (2.33)

The mean values shown are averages are from 3 independent replicates

*p < 0.001 compared to first passage through resin

### Effectiveness of subsequent use of Pierce™ high-capacity endotoxin removal Resin spin columns

**Fig. 4:**
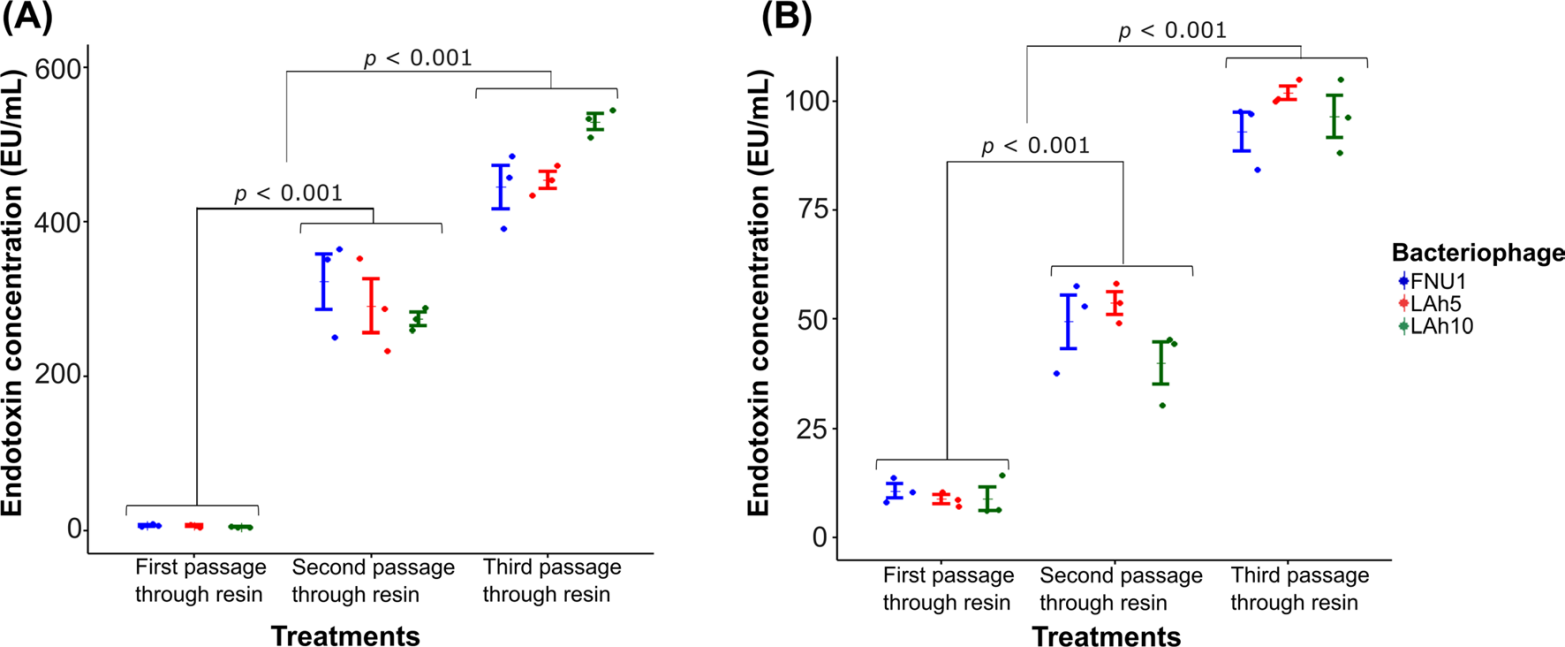
Estimated endotoxin concentrations of purified bacteriophage preparation with the Pierce™ High-Capacity Endotoxin Removal Resin spin columns were calculated from standard curves: **(A)** TNF-α and **(B)** IL-6. Wilcoxon signed-rank test was used to analyse the data (*n* = 3; error bars indicate standard error of the mean; *p*  < 0.05)

Pierce™ High-Capacity Endotoxin Removal Resin spin columns are considered multi-use products [30]. In this study, the columns were used three times, each time with a new crude sample, to determine whether these columns were still as effective in removing endotoxins. Estimated endotoxin concentration of purified bacteriophage preparations using the columns was lowest when they were used the first time, *p* < 0.001 (Table 4). When columns were used for the third time, the endotoxin concentration was significantly higher than the second use, *p* < 0.001 (Fig. 4), although this is still an improvement of nearly a 3,000-fold decrease in endotoxin concentration compared to the crude preparations (Table 2).

### Bacteriophage viability following purification

**Fig. 5:**
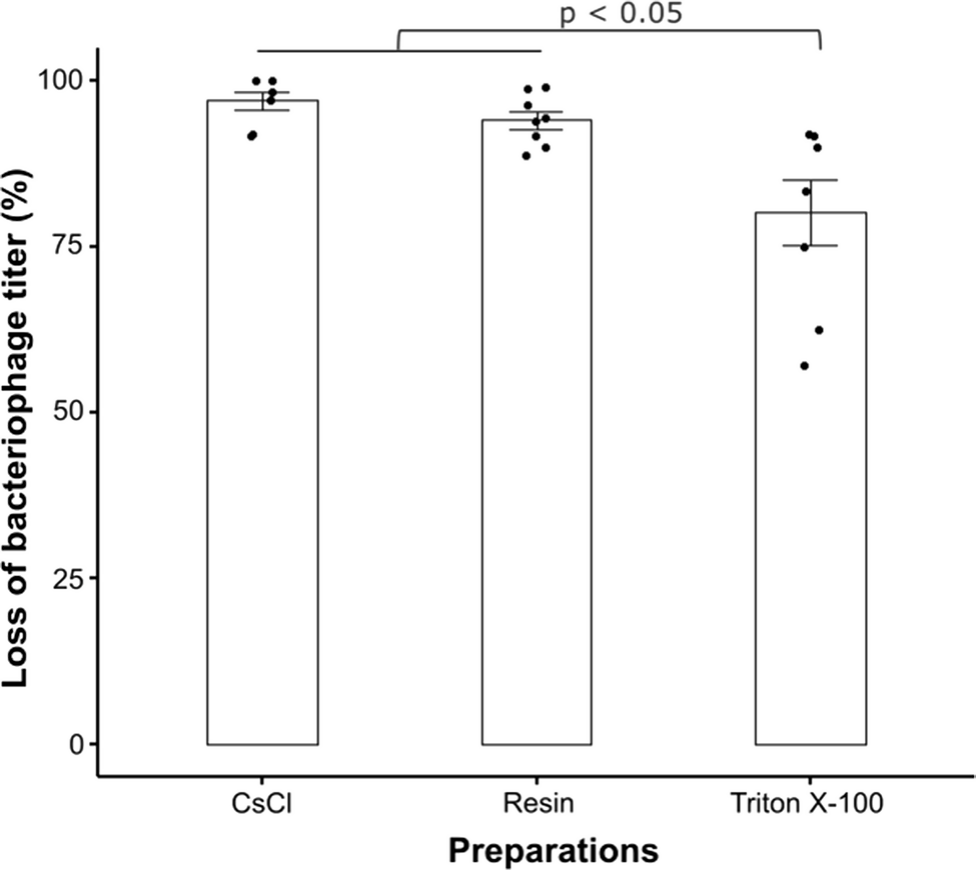
Percentage of loss of bacteriophage titre after purification. Bacteriophage concentrations were calculated in PFU/mL before and after purification. Titre loss was calculated for each bacteriophage purification method. Wilcoxon signed-rank test was used to analyse the data (*n* = 8; *p* < 0.05)

The recovered bacteriophages were assayed following each of the purification techniques to determine any loss in bacteriophage titre. Bacteriophages purified using CsCl ultracentrifugation or the Pierce™ High-Capacity Endotoxin Removal Resin spin columns lost approximately 90% of their titre, a significantly higher loss than bacteriophage preparations purified using Triton X-100 (average of 71% loss of titre) (Fig. 5). Final concentrations of bacteriophages after purifications are listed in the supplementary Table S1.

## Discussion

Three bacteriophage morphotypes were purified using three common techniques to determine the efficacy of purification methods in reducing monocyte response to bacteriophage preparations. All three purification methods used in this study reduced endotoxin levels regardless of morphology and size of the virus. Triton X-100 was optimal at removing endotoxins and retaining bacteriophage titre. CsCl ultracentrifugation had comparable efficacy in reducing endotoxin but with approximately 20% lower recovery rate of bacteriophages. The Pierce™ High-Capacity Endotoxin Removal Resin spin columns were the least effective in removing endotoxin, and resulted in similar loss of bacteriophage titre as CsCl ultracentrifugation.

In measuring endotoxin concentrations, commercially available amoebocyte lysate assays and wild-type monocyte assays are limited to detecting less than 1 EU mL^− 1^ of endotoxin. In comparison, the IRAK3 knockout monocyte assay we developed and employed here was able to detect a broader range of endotoxin concentrations. This may limit measurement errors that are possibly introduced if samples need to be serially diluted for measurement with commercial kits. When we tested a broad range of concentrations of endotoxin standards, the IRAK3 knockout monocytes produced a biphasic cytokine response. The biphasic response revealed strong correlation between the production of IL-6 or TNF-α and endotoxin concentration. It is unclear why there was a biphasic response. Other mediators of cytokine release impact responses to endotoxin as IRAK3 deletion in human primary monocytes or THP-1 cells results in upregulation of multiple families of cytokines [31]. Of the two cytokines, IL-6 had a better correlation for the low concentration standards, while TNF-α had a better correlation for the high concentration standards. Both cytokines are therefore key in establishing accurate endotoxin concentrations in bacteriophage preparations using our assay.

The production of pro-inflammatory cytokines TNF-α and IL-6 was significantly higher in the crude bacteriophage preparations compared to the purified preparations for all methods tested. Endotoxin concentrations were significantly lower for Triton X-100 purification, compared to CsCl ultracentrifugation, when measured via TNF-α production (which has a lower correlation coefficient) from the standard curve using low concentrations. When using IL-6 production as a measure, bacteriophage preparations purified with Triton X-100 did not differ to those purified with CsCl ultracentrifugation. These results suggest that bacteriophage purification with Triton X-100 was at least as effective as CsCl ultracentrifugation without the need for expensive equipment such as ultracentrifuges. Previous studies have used at least two sequential methods [17, 18] to effectively remove endotoxins from bacteriophage preparations. We found that single-step purifications with Triton X-100 or CsCl ultracentrifugation reduced endotoxin concentration to levels comparable to those of the sequential techniques [17, 18].

The efficacy of the purification techniques was similar for the three different bacteriophages tested. These differed in capsid size, genome size and morphology (representing the three common morphotypes of siphovirus, myovirus and podovirus). Although some chemicals are known to affect bacteriophages of different morphology differently [32, 33], we did not see any variances in purification efficacy here.

There have been suggestions that purification methods such as Triton X-100 and CsCl, result in remnant chemicals rendering bacteriophage preparations not safe for clinical use [7]. Previous studies have shown that CsCl may suppress the growth of HeLa cells [34] and has been associated with chromosomal aberrations in mice [35, 36]. Similarly, while some Triton X detergents are well tolerated [37], low concentrations of Triton X-100 may lead to cell death, raising concerns about use in preparation of therapeutics [38, 39]. On occasion, clinical studies have included additional steps such as dialysis in PBS are employed to remove these harmful chemicals before administering to patients [40–42]. In our study, three PBS wash steps were employed to minimise remnant Triton X-100 in purified bacteriophage preparations. Using this method, we have shown that the resulting bacteriophage preparations do not adversely affect growth of human epithelial cells [27]. It is also important to note that bacteriophage purification using these methods is not new, and that endotoxin purifications using CsCl have been applied for many years and considered generally safe [7].

Bacteriophage preparations purified using Pierce™ High-Capacity Endotoxin Removal Resin spin columns reduced endotoxin levels significantly compared to crude preparations, but the levels were significantly higher than both CsCl ultracentrifugation and Triton X-100 preparations. Although promoted as a reusable technique, the spin column was most effective in removing endotoxin in bacteriophage preparations during the first use. Each subsequent reuse of the spin columns was less effective in removing endotoxins. This is similar to findings observed by Hietala et al. [18]. It is possible that high endotoxin concentrations in crude bacteriophage preparations caused resin saturation after each use and the equilibration steps did not allow dissociation of bound endotoxin. The bacteriophage titre loss in resin purified bacteriophages was similar to CsCl ultracentrifugation, at approximately 90%.

We [19, 43] and others [44–46] have previously shown that purified bacteriophages induce low levels of cytokine production. This minimal immune response induced by the purified bacteriophages illustrates that they may be a safe option when administered in therapy. However, studies have also reported that bacteriophage specific antibodies are present after exposure [47], and it remains unclear whether this is a result of inadequately purified bacteriophages that activate the innate and subsequent adaptive immunity. Another complicating factor is that innate responses to bacteria can affect responses to bacteriophages [48].

## Conclusions

In this study, Triton X-100 was the most effective method for bacteriophage recovery. Although we found lower retention of viable bacteriophages when using CsCl, endotoxin removal efficacy was comparable to Triton X-100. Both these methods were significantly more effective than resins in removing endotoxins. Further, differences in the morphology, capsid size or genomic size of the bacteriophages used in this study did not influence the efficacy of bacteriophage purification.

## Electronic supplementary material

Below is the link to the electronic supplementary material.

Supplementary Material 1

## Data Availability

No datasets were generated or analysed during the current study.

## Abbreviations

CO_2_: Carbon Dioxide
DNA: Deoxyribonucleic Acid
EIA: Enzyme Immuno-Assay
ELISA: Enzyme-Linked Immunosorbent Assay
EU: European Union
FDA: Food and Drug Administration
IL: Interleukin
IRAK3: Interleukin Receptor-Associated Kinase 3
LAL: Limulus Amoebocyte Lysate
LPS: Lipopolysaccharide
PBS: Phosphate Buffered Saline
PCR: Polymerase Chain Reaction
PEG: Polyethylene Glycol
PFU: Plaque Forming Unit
RCT: Randomised controlled trial
RNA: Ribonucleic acid
RPMI: Roswell Park Memorial Institute
TNF - α: Tumour necrosis factor - α
