# Supplementary material for: Evaluation of effectiveness of bacteriophage purification methods
Source: Virol J. 2024 Dec 19;21:318. doi: 10.1186/s12985-024-02580-y (PMC11656862; doi:10.1186/s12985-024-02580-y)
Supplement: Supplementary file 1 — Supplementary Material 1 [file 12985_2024_2580_MOESM1_ESM.docx]

**Supplementary data**

| **Table S1.** Summary data for bacteriophage concentrations after each purification method from 1×10^9^ PFU mL^-1^. | | | |
| --- | --- | --- | --- |
| **Purification method** | **Bacteriophage** | **Average bacteriophage (PFU mL^-1^)** |  |
| Triton X-100 | FNU1 | (3 ± 0.82) × 10^8^ |  |
|  | LAh5 | (3 ± 1.63) × 10^8^ |  |
|  | LAh10 | (2.70 ± 2.01) × 10^8^ |  |
| CsCl | FNU1 | (5.67 ± 1.07) × 10^4^ |  |
|  | LAh5 | (6.00 ± 2.16) × 10^7^ |  |
|  | LAh10 | (1.17 ± 0.62) × 10^8^ |  |
| Resin | FNU1 | (6.00 ± 2.16) × 10^7^ |  |
|  | LAh5 | (3.27 ± 4.05) × 10^8^ |  |
|  | LAh10 | (3.67 ± 2.49) × 10^7^ |  |
